# Case report: Autoimmune encephalitis with multiple auto-antibodies with reversible splenial lesion syndrome and bilateral ovarian teratoma

**DOI:** 10.3389/fimmu.2022.1029294

**Published:** 2023-01-12

**Authors:** Yaqiang Li, Mei Zhang, Deshun Liu, Ming Wei, Jun Sheng, Zhixin Wang, Song Xue, Tingting Yu, Weimin Xue, Beibei Zhu, Jiale He

**Affiliations:** ^1^ Department of Neurology, First Affiliated Hospital of Anhui University of Science and Technology (First People’s Hospital of Huainan), Huainan, China; ^2^ Department of Neurology, People’s Hospital of Lixin County, Bozhou, China; ^3^ Department of Radiology, First Affiliated Hospital of Anhui University of Science and Technology (First People’s Hospital of Huainan), Huainan, China; ^4^ Department of Gynecology and Obstetrics, First Affiliated Hospital of Anhui University of Science and Technology (First People’s Hospital of Huainan), Huainan, China; ^5^ Department of Pathology, First Affiliated Hospital of Anhui University of Science and Technology (First People’s Hospital of Huainan), Huainan, China

**Keywords:** reversible splenial lesion syndrome, autoimmune encephalitis, ovarian teratomas, anti-N-methyl-D-aspartame receptor (anti-NMDAR) encephalitis, anti-metabotropic glutamate receptor 5 (mGluR5) encephalitis

## Abstract

**Background:**

Reversible splenial lesion syndrome (RESLES) is a spectrum of disease radiologically characterized by reversible lesions caused by multiple factors, primarily involving the splenium of the corpus callosum (SCC). The most common causes of RESLES include infection, antiepileptic drug use and withdrawal, and severe metabolic disorders. Nevertheless, cases of autoimmune encephalitis (AE) are uncommon.

**Case presentation:**

A 26-year-old female computer programming engineer with no previous medical or psychiatric history reported to the psychiatric hospital due to a 3-day episode of irritability, babbling, limb stiffness, sleepwalking, hallucinations, and paroxysmal mania. Brain MRI revealed abnormal signals of the SCC. Lumbar puncture was performed and further testing for auto-antibodies was conducted in both the CSF and serum. CSF of the patient was positive for anti-NMDAR (titer of 1:3.2) antibodies, and serum was also positive for anti-NMDAR (titer of 1:32) as well as mGluR5 (titer of 1:10) antibodies. Enhanced CT of the pelvis showed an enlarged pelvic mass; bilateral ovarian teratomas (mature teratoma and immature teratoma) were evaluated, which were pathologically confirmed after transabdominal left adnexal resection, right ovarian biopsy, and ovarian cystectomy. The patient considerably improved after intravenous administration of steroids, immunoglobulin, oral prednisone, surgical treatment, and chemotherapy. A follow-up MRI revealed completely resolved lesions. During a 3-month follow-up, the patient experienced complete resolution of symptoms without any sign of recurrence and tumors. The titer of the anti-NMDAR antibody decreased to 1:10 in serum.

**Conclusion:**

Herein, we report a rare case of AE with overlapping auto-antibodies, along with RESLES and bilateral ovarian teratomas. The current case provides the possibility of the concurrence of mGluR5 antibodies in anti-NMDAR encephalitis. However, the underlying mechanism remains elusive. Furthermore, we provide additional evidence that overlapping antibodies-related pathology may be one of the many causes of RESLES. Nonetheless, caution should be observed in interpreting the observation, considering that this is a single-case study.

## Introduction

Anti-N-methyl-D-aspartame receptor (anti-NMDAR) encephalitis is the most commonly known type of autoimmune encephalitis (AE), accounting for 6% to 10% of all encephalitis. It is characterized by severe neuropsychiatric manifestations, particularly psychiatric symptoms (1–3). Unlike other AE, the disease is characterized by the presence of an IgG1 antibody directed against the NR1 subunit of the NMDAR on the neuronal cell surface or synapses in the cerebrospinal fluid (CSF); however, 14.4% to 28.6% of patients have a negative serum antibody test (4, 5). Other auto-antibodies, including anti-alpha-amino-3-hydroxy-5-methyl-4-isoxazolepropionic acid receptor (AMPAR), anti-leucine rich glioma inactivated 1 (LGI1), anti-gamma-aminobutyric-acid B receptor (GABABR), and anti-contactin-associated protein 2 (CASPR2) antibodies are also common (6–9). The occurrence of mGluR5 auto-antibodies was initially reported in 2 young patients with Ophelia syndrome, an encephalitis associated with Hodgkin’s lymphoma characterized by psychosis, memory deficits, and a dreamy state (10). Of note, co-existing antibodies may produce an overlap of clinical symptoms in patients with AE, increasing the probability of potential malignancy (11). Literature reports indicate that anti-NMDAR encephalitis occurs mostly in women with an average onset age of approximately 21 years and is frequently related to tumors, especially ovarian teratomas (12–14). A recent systematic review of anti-NMDAR encephalitis found that the most common brain magnetic resonance imaging (MRI) outcome was abnormal temporal lobe signals in the medial temporal lobe with hyperintensity on fluid-attenuated inversion recovery (FLAIR), followed by cortical gray matter changes and subcortical white matter changes. Furthermore, soft meningeal enhancement, followed by cortical enhancement was the most common dynamic contrast-enhanced MRI outcome (15). Reversible splenial lesion syndrome (RESLES) is a clinico-radiological syndrome characterized by reversible lesions in the splenium of corpus callosum (SCC) and commonly caused by infection, antiepileptic drugs use, antiepileptic drugs withdrawal, and severe metabolic disorders (16). Overlapping auto-antibodies and RESLES rarely occur together in AE. Here, we report a rare case of AE with anti-NMDAR as well as anti-mGluR5 antibodies in the serum and anti-NMDAR only in the CSF with RESLES and bilateral ovarian teratomas.

## Case presentation

**Figure 1:**
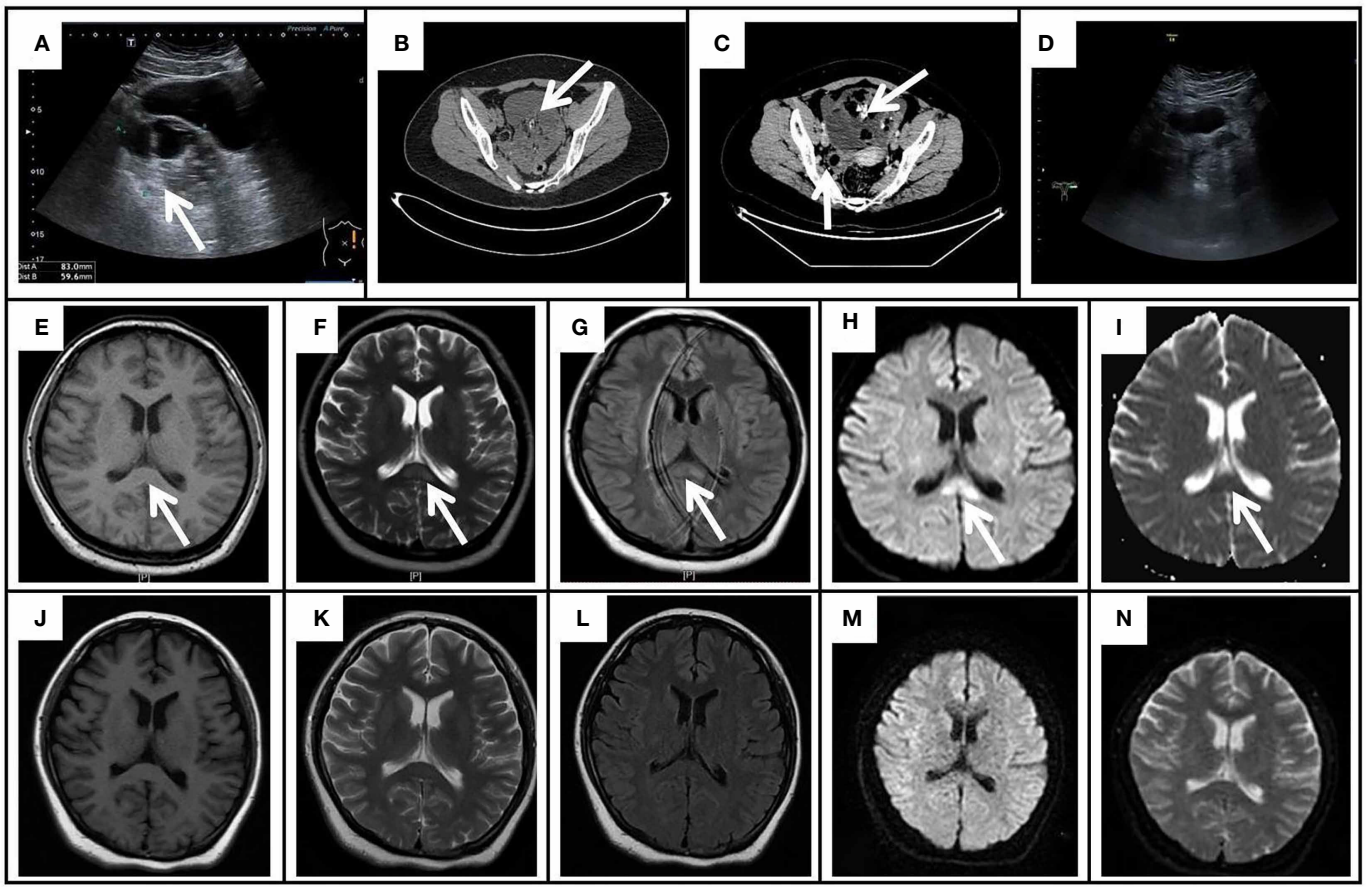
The gynecological ultrasound, pelvic CT, and cranial MRI results of the patient. **(A)** The gynecological ultrasound showed a mixed echogenic mass (8.3 cm × 5.9 cm) in the left ovary. **(B)** Pelvic CT showed an irregular cystic mass (10.2 cm × 8.4 cm) with a few calcifications and fatty components in the posterior aspect of the bladder. **(C)** Enhanced CT of the pelvis revealed a large cystic-solid mass in the anterior aspect of the uterus with no enhancement of the cystic component, fat and calcification, and mild enhancement of the solid component; A mass of uneven density on the right side of the uterus with a predominantly solid component, mild enhancement of the solid component and no enhancement of the fatty component. **(D)** The repeated gynecological ultrasound demonstrated no abnormal echogenicity. Initial cranial MRI exhibited an isolated splenium of the corpus callosum (SCC) lesion (arrows) with slight hypointensity on T1WI **(E)**, hyperintensity on T2WI **(F)**, FLAIR **(G)**, and DWI **(H)**, and hypointensity on the ADC map **(I)**. On day 52 after onset, repeated MRI demonstrated the lesion of SCC lesion was completely resolved on T1WI **(J)**, T2WI **(K)**, FLAIR **(L)**, DWI **(M)**, and ADC **(N)**.

**Figure 2:**
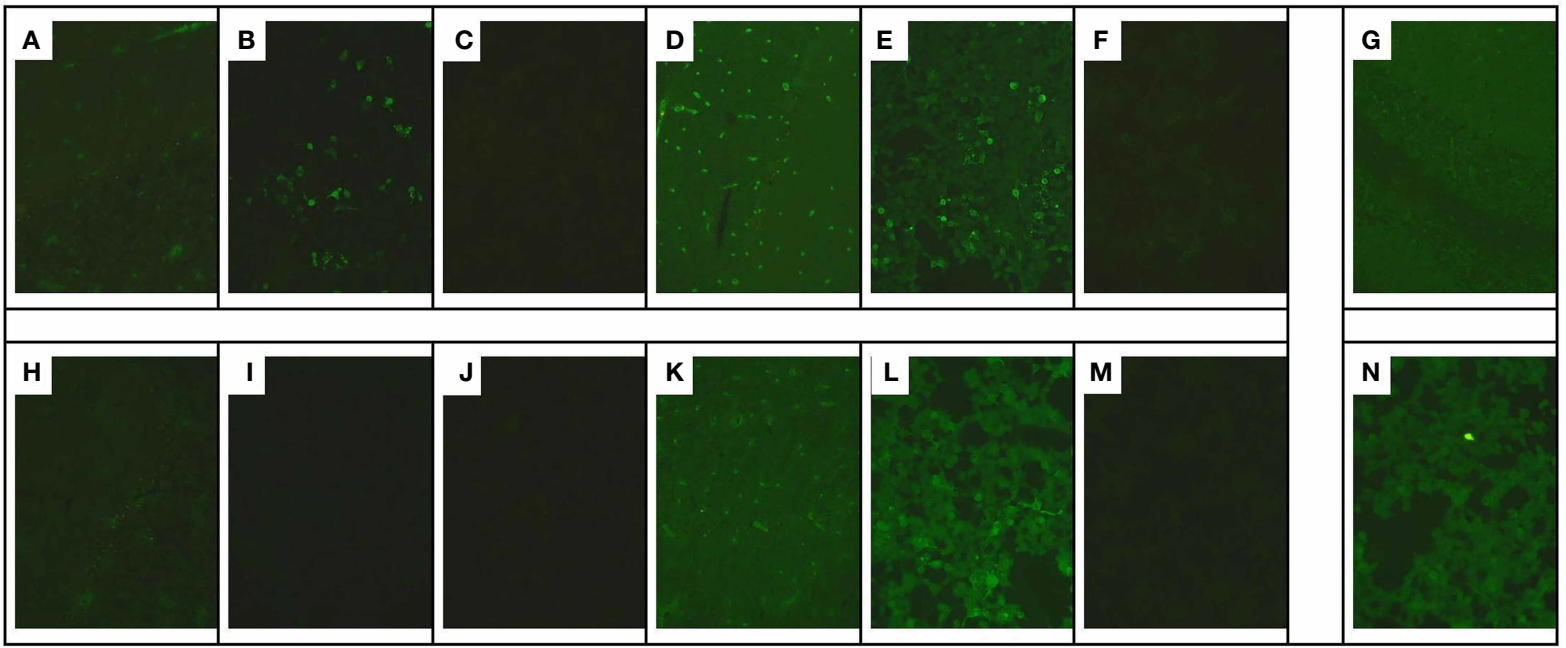
Immunofluorescence staining of monkey cerebellar tissues. Cryosections of the monkey cerebellum were incubated with patient serum and CSF in the first step and with FITC-labeled goat anti-human IgG in the second step. Immunofluorescence of anti-NMDAR and anti-mGluR5 antibodies in the CSF and serum of the patient. Anti-mGluR5 and anti-NMDAR antibodies bound on mGluR5 and NMDAR antigens expressed in HEK293 cells, respectively, and visualized by the immunofluorescence of fluorescein on the second antibody. **(A)** Tissue-based indirect immunofluorescence assay of CSF displayed anti-NMDAR antibody presenting as granular fluorescence in monkey cerebellar granular layer cells. ×200 magnification. **(B)** Positive reaction with transfected HEK293 cells expressing NMDAR after incubation with the patient’s first CSF **(B)** (titer 1:3.2). ×200 magnification. **(C, I)** Negative reaction with transfected HEK293 cells expressing mGluR5 after incubation with the patient’s first CSF **(C)** and the repeated CSF **(I)**. ×200 magnification. **(D, G, J, M)** Tissue-based indirect immunofluorescence assay of the patient’s first serum **(D)**, second CSF **(G)**, second serum **(J)**, and third serum **(M)** showed no specific form of fluorescence in the monkey cerebellum. **(E, K, N)** Positive reaction with transfected HEK293 cells expressing NMDAR after incubation with the patient’s first serum (titer 1:32) **(E)**, second serum (titer 1:32) **(K)**, and third serum (titer 1:10) **(N)**. **(F)** Positive reaction with transfected HEK293 cells expressing mGluR5 after incubation with the patient’s first serum **(F)** (titer 1:10). ×200 magnification. **(H)** Negative reaction with transfected HEK293 cells expressing mGluR5 after incubation with the repeated CSF **(H)**. ×200 magnification. **(L)** Negative reaction with transfected HEK293 cells expressing mGluR5 after incubation with the repeated serum **(L)**. ×200 magnification.

A 26-year-old female computer programming engineer with no previous medical or psychiatric history visited the psychiatric hospital on December 4, 2021, following a 3-day episode characterized by irritability, babbling, stiffness of the limbs, sleepwalking, hallucinations and paroxysmal mania, all of which had a significant impact on her life. The parents of the patient attributed the episode partially to her recent work stress and breakup. The patient had no history of headache, dizziness, fever, seizures, and dyskinesia, and was diagnosed with schizophrenia by a psychiatrist. During hospitalization, the patient was prescribed olanzapine 5mg twice daily, and sertraline 50mg once daily, but the symptoms were poorly controlled. On the 8th day after initial symptoms, a head computed tomography (CT) scan was performed, and no significant abnormalities were observed. An electroencephalogram (EEG) revealed a diffused slow wave. On the 17th day after initial symptoms, the abnormal signals of the SCC were characterized by slight hypo-intensity on T1-weighted image (T1WI), hyper-intensity on T2-weighted image (T2WI), FLAIR, and diffusion-weighted imaging (DWI) imaging, and hypo-intensity on apparent diffusion coefficient (ADC) map (**Figures 1E–I**). Despite the treatment, the condition of the patient progressed and was transferred to the department of the neurological department on the 20th day after initial symptoms (Admission Day). Neurological examination showed drowsiness, difficulty with attention, or difficulty with serial 7s. The muscle strength of her limbs was approximately graded 5/5, with normal muscle tone and tendon reflexes; the remaining physical and neurological examination results were not significant. On admission day, a lumbar puncture was performed, showing homogeneous and uniform light red CSF in the anterior and posterior canals, normal opening pressure and glucose levels, increased CSF protein levels (1195.4 mg/L), normal white cell count (4×10^6/L), and high red blood cell count (1.2×10^9/L). After centrifugation, the supernatant of the CSF remained light red, and was positive for an occult blood test; crenocytes could be observed under a light microscope (×800 magnification) (**Supplementary Material**). No abnormalities were observed in the CSF bacteriological tests, CSF Gram stain, and CSF virus tests (herpes simplex virus, varicella-zoster virus, enterovirus, cytomegalovirus, rubella virus, and Epstein-Barr virus). Furthermore, we performed additional hospital-related examinations, including a coagulation function test. As a consequence, the mild elevation of D-Dimer was 2.93 mg/L (normal range, 0-0.55 mg/L) and the mild elevation of fibrinogen degradation products was 7.58 mg/L (normal range, 0-5 mg/L). Besides, alanine aminotransferase 56 U/L (normal range, 8-40 U/L), other biochemical indicators, complete blood cell count (including a white blood cell count of 8.5×10^6/L and red blood cell count of 5×10^12/L), routine urine and stool tests, homocysteine, thyroid-stimulating hormone, antithyroglobulin, antithyroperoxidase antibodies, cortisol, vitamin B12, erythrocyte sedimentation rate, and female tumor markers were normal. Serology for syphilis, hepatitis B, mycoplasma, human immunodeficiency virus antibody, herpes simplex virus, hepatitis C virus antibody, varicella, cytomegalovirus, mumps, measles, and Epstein-Barr virus was negative. Moreover, anti-nuclear, anti-SM, anti-SSB, anti-double-stranded DNA, anti-SSA, anti-JO-1, and all other auto-antibody tests were negative. Considering the probability of AE, an antibody test of CSF using a cell-based assay combined with a tissue-based assay (EUROIMMUN Medical Diagnostics company) was also performed on the second day of admission (**Figures 2A–F**). The anti-NMDAR (titer of 1:3.2) antibody was positive. Serum analysis revealed positive anti-NMDAR (titer of 1:32), and anti-mGluR5 (titer of 1:10) antibodies, but negative anti-AMPA1, anti-AMPA2, anti-GABA-B, anti-LGI 1, anti-GAD65, anti-CASPR2, anti-DPPX, and anti-IgLON5 antibodies. In addition, CNS demyelinating antibodies, including AQP4, MOG, and MBP were also negative. Given the concern for AE, the patient was administered intravenous methylprednisolone (1 g per day, intravenously, for 5 days, 500 mg per day for 3 days, 250 mg per day for 3 days) and intravenous immunoglobulin (0.4 g/kg body weight for 5 days) on the 2nd day after admission. At the same time, olanzapine improved psychiatric symptoms. After 5 days of treatment, the symptoms gradually improved, her mental state improved, and could communicate. One month after the initial symptoms, the patient had not experienced sleepwalking, her mental and behavioral symptoms had largely disappeared, her memory had gradually recovered, and her arithmetic skills had improved significantly unlike before. The Mini-Mental State Examination (MMSE) scores and Montreal Cognitive Assessment (MoCA) scores were 22 and 18, respectively. We further examined for tumor evidence and a gynecological ultrasound revealed a mixed echogenic mass in the left ovary (**Figure 1A**). Malignancy screening, including a CT of the chest, abdomen, and pelvis was performed to further assess the tumors, which demonstrated an irregular cystic mass (10.2 cm × 8.4 cm) with a few calcifications and fatty components in the posterior region of the bladder; ovarian teratoma was suspected (**Figure 1B**). Subsequently, a gynecologist recommended surgical removal of the pelvic mass, however, the patient temporarily postponed the surgical treatment for personal reasons, and thus was discharged from the hospital on the 19nd day after admission. At this time, the patient had transitioned to prednisone 60 mg, reduced by 5 mg every 2 weeks, with anticipated treatment lasting 6 months. At 1 month follow-up, her mental and behavioral symptoms improved completely, with a score of 30 for both MMSE score and MoCA scores. A follow-up MRI scan (**Figures 1J–N**) indicated that the lesion in SCC had completely resolved 2 weeks after discharge.

The patient was admitted to our gynecology department on May 16, 2022 (Admission Day), due to intermittent pain in the left lower abdomen for 3 months. A systematic abdominal and neurological examination was performed, revealing abdominal distension, increased abdominal wall tension, and a palpable abdominal mass on the lower abdominal surface; the remaining physical and neurological examination results were not significant. After admission, relevant examinations were performed again. No abnormality was detected by routine blood, urine, and stool tests, biochemical indicators, coagulation, and thyroid functions. Tumor markers were investigated on the 2nd day after admission, and the results revealed alpha-fetoprotein of 51.6 ng/ml (normal range, 0-20 ng/ml) and carbohydrate antigen 125 of 266.8 U/ml (normal range, 0-35 U/ml).

Furthermore, malignancy screening, including the enhanced CT of the abdomen and the pelvic mass was performed to re-evaluate the tumors on the 4nd day after admission. Enhanced CT of the pelvis revealed: a large cystic-solid mass (19 cm × 11 cm) in the anterior region of the uterus with no enhancement of the cystic component, fat and calcification, and mild enhancement of the solid component; A mass (3.3 cm × 2.9 cm) of uneven density on the right side of the uterus with a predominantly solid component, mild enhancement of the solid component and no enhancement of the fatty component (**Figure 1C**). Transabdominal left adnexal resection, right ovarian biopsy, and ovarian cystectomy were performed under general anesthesia on the 9nd day after admission.

**Figure 3:**
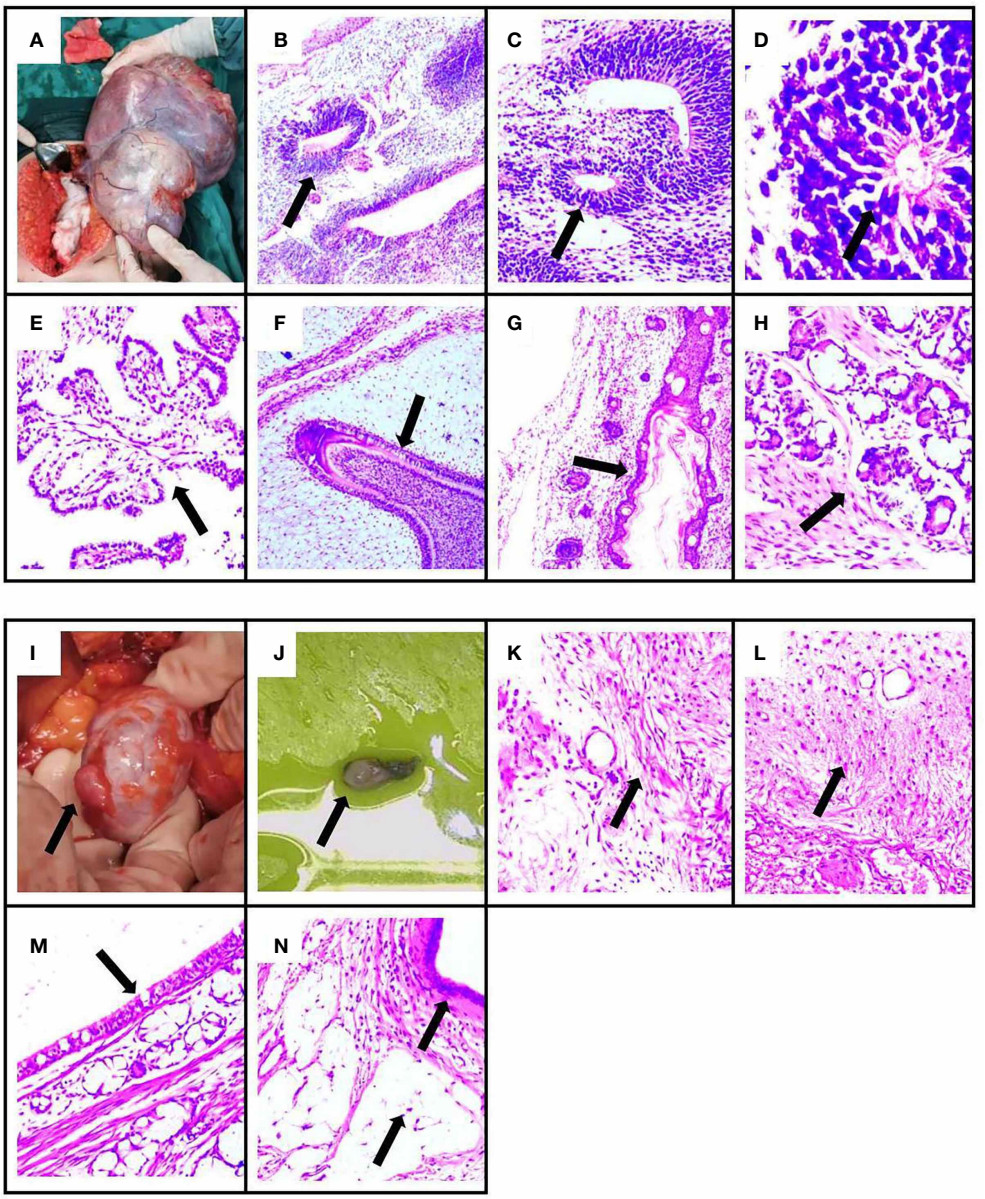
The pathology results and surgical specimen of the patient. The specimen showed immature ovarian teratoma, consisting of neuroglia, primitive neural tube, infantile neural tissue, choroid plexus tissue, naive enamel organ, epidermis, hair follicle, and salivary gland tissue by H&E staining. The arrows indicate lesions in **(B–H)**. **(B)** Neuroglia. **(C)** Primitive neural tube. **(D)** Infantile neural tissue. **(E)** Choroid plexus tissue. **(F)** Naive enamel organ. **(G)** Epidermis and hair follicle. **(H)** Salivary gland tissue. **(A)** The immature ovarian teratoma after surgical resection. The specimen showed mature ovarian teratoma, consisting of mature neuroglial, mature brain tissue, respiratory epithelium, mature adipose tissue, and keratocysts by H&E staining. The arrows indicate lesions in **(K–N)**. **(K)** Mature neuroglial. **(L)** Mature brain tissue. **(M)** Respiratory epithelium. **(N)** Mature adipose tissue and keratocysts. **(I, J)** The mature ovarian teratoma after surgical resection.

**Figure 4:**
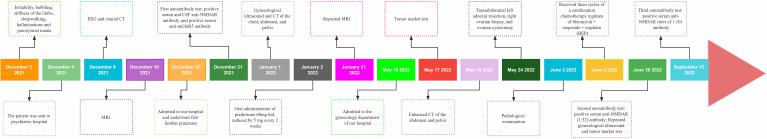
Timeline with clinical manifestation, treatment progression, and diagnosis time.

Intraoperatively, irregular tissue of the left adnexa was observed, with a size of approximately 27*21*7 cm (**Figure 3A**), and the right ovary was slightly enlarged; a papillary bulge was visible on the surface of the ovary, with a size of approximately 1*0.5*0.5 cm (**Figures 3I, J**). The pathology report showed: immature cystic teratoma (histological grade 2, clinical stage IC) in the left ovary, comprising neuroglia, primitive neural tube, Infantile neural tissue, choroid plexus tissue, naive enamel organ, epidermis, hair follicle and salivary gland tissue (**Figures 3B–H**); Mature cystic teratoma in the right ovary, consisting of mature neuroglial, mature brain tissue, respiratory epithelium, mature adipose tissue and keratocysts (**Figures 3K–N**). Surgical intervention was followed by three cycles of a combined chemotherapy regimen of bleomycin + etoposide + cisplatin (BEP) on June 4^th^. The patients achieved complete resolution of abdominal pain without any sign of recurrence and tumors during a 1-month follow-up. Tumor markers revealed CA125 of 22.2 U/ml (normal range, 0-35 U/ml) and AFP of 1.8/μl (normal range, 0-20ng/ml). The gynecological ultrasound demonstrated no abnormal echogenicity (**Figure 1D**). Anti-mGluR5 antibody in the serum and anti-NMDAR antibody in the CSF were negative, respectively, and the titer of the anti-NMDAR antibody in the serum remained 1:32 (**Figures 2G–L**). At the 3-month follow-up, although her titer of anti-NMDAR antibodies in serum had decreased to 1:10 (**Figures 2M, N**), her daily life was not significantly affected and she resumed work. The entire course of the disease and the therapeutic modalities used have been summarized as a graphical abstract (**Figure 4**).

## Discussion

For the first time, we report a rare case of a patient diagnosed with overlapping auto-antibody syndromes with RESLES and bilateral ovarian teratoma. The patient was a young woman, with abnormal behavior (hallucinations and agitation) with irritability, followed by speech dysfunction, dyskinesias, and a drop in consciousness level. Based on positive anti-NMDAR antibodies in both CSF and serum, this case satisfied the criteria of definite anti-NMDAR encephalitis (17). So far, few reports exist on mGluR5-associated encephalitis; only 13 cases of mGluR5-associated encephalitis have been reported, with main clinical manifestations comprising abnormal psychiatric behavior, cognitive impairment, sleep disturbance, seizures, decreased level of consciousness, and movement disorder (10, 18–21). In the present case, the clinical manifestations of psychiatric and cognitive disorders could be simultaneously attributed to anti-mGluR5 and anti-NMDAR antibodies. Sleepwalking could be associated with anti-GluR5 antibodies. The presence of mGluR5 in anti-NMDAR encephalitis suggests the possibility of an overlapping autoimmune complex. Thus far, no case report of the simultaneous presence of the anti-mGluR5 and anti-NMDAR antibodies similar to our case has been published in PubMed.

Glutamate is a major excitatory neurotransmitter of the central nervous system and modulates many key neuronal functions (22). However, glutamate overload can cause massive neuronal death and excitatory brain damage due to excessive glutamate receptor activation. Glutamate receptors (GluRs) are primarily classified into ionotropic (iGluRs), which act as glutamate-gated ion channels, and metabotropic (mGluRs), which are G protein-coupled receptors that span seven transmembranes, coupling to G proteins and activating intracellular signaling (23, 24). NMDARs are ligand-gated cation channels that regulate synaptic transmission and plasticity. They are localized in the postsynaptic membrane of neurons and predominantly found in the forebrain and limbic system, specifically the hippocampus. The pathogenesis of anti-NMDAR encephalitis may be attributed to antibody cross-linking and capping and internalization of NMDARs, causing decreased receptor density and reduced synaptic function of neurons (25, 26). mGluR5 is mainly found in the postsynaptic terminals of neurons and microglia. mGluR5 signals through Gq/G11 coupling to activate phospholipase C, leading to calcium mobilization and activation of protein kinase C, mainly expressed in the hippocampus and amygdala. These antibodies cause a decrease in mGluR5 cluster density at both synaptic and extrasynaptic locations, although the exact mechanism by which these antibodies alter receptor density remains unknown (20). Notably, glutamate exerts its important effects through mGluR5s and NMDARs. Antibodies against these two receptors cause anti-NMDA receptor encephalitis and anti-mGluR5 receptor encephalitis, both of which can result in overlapping auto-antibody syndromes.

Autoimmune antibodies can be divided into two types, i.e ., (1) antibodies directed against intracellular proteins (e.g. anti-Hu, Yo, and Ri antibodies) ; (2) antibodies acting on neuronal cell-surface receptors or synaptic proteins (e.g. anti-NMDAR, mGluR5, GABABR, LGI1, and AMPAP antibodies), some of which can occasionally overlap with each other, such as the combination of anti-NMDAR antibodies with anti-GABABR antibodies, anti-NMDAR antibodies with anti-IGI1 antibodies, and anti-NMDAR antibodies with anti-AMPAP antibodies (12, 27, 28). Of note, it is unclear why both antibodies are present in our patient, given their uncommon coexistence. The propensity for overlapping autoantibody syndromes also needs further exploration to elucidate whether either or both antibodies are pathogenic.

The clinical features of overlapping auto-antibody neurological syndromes may be common for each antibody-mediated disease, which individually combine into a single, distinct presentation that is atypical for any one antibody. For example, patients with anti-mGluR5 antibodies combined with positive anti-SOX1 antibodies have symptoms of cerebellar ataxia (20). Hoftberger et al. (27) reported a case of anti-GABABR encephalitis combined with positive anti-NMDAR antibodies, in which the psychiatric symptoms were more pronounced. They reported a similar case but combined with positive anti-GAD65 antibodies accompanied by refractory epilepsy. The titer of anti-mGluR5 antibodies in our case history was low and was only found in serum. It is unlikely that they represented true neurological autoimmunity in this situation. Nevertheless, it may be a marker of increased auto-antibodies produced in response to tumor cell breakdown and antigen release or represents a low titer “false positive” result, as seen in 8% of the general population (29). Since patient serum samples were evaluated three times and the serum anti-NMDAR antibodies remained positive during follow-up with decreasing antibody titers, we hypothesized a relatively low likelihood of a false-positive result for anti-NMDAR antibodies.

The initial brain MRI showed an abnormal signal in the splenium of the corpus callosum (SCC) and repeated MRI revealed complete resolution of the lesion, and the case met the diagnostic criteria for RESLES (16). RESLES commonly occur in children and young adults due to a number of factors, including metabolic disorders, high altitude cerebral edema, infections, and rare causes such as Kawasaki disease, acute poisoning, vitamin B12 deficiency, systemic lupus erythematosus, anorexia nervosa and cerebral venous thrombosis (30–33). Besides, it is commonly reported in patients under antiepileptic drugs, especially after drug withdrawal (16). However, the patient had no previous history of epilepsy, no epileptoid seizure, vomiting, and other symptoms during the onset of the disease, as well as no disorders including hypoglycemia, malnutrition, and liver or kidney failure. Therefore, epilepsy and metabolic disorders (hyponatremia, hypoglycemia, nutritional deficiency, etc.) could not have caused RESLES. Furthermore, the patient had no fever or other signs of infection, and microbiological tests in serum and CSF were negative; therefore, the probability of viral or other pathogenic infection was low. In addition, demyelinating antibodies in the CSF, biochemical indicators, vitamin B12 levels, thyroid function, and rheumatic function tests showed normal metabolic function and no other signs of autoimmune or demyelinating diseases. Repeated MRI revealed complete recovery of the lesion, thus excluding ischemic stroke.

The causative mechanism of RESLES is unclear. It is generally considered to be transient cytotoxic edema of SCC caused by various etiologies, which can be inferred by restricted diffusion on DWI and low ADC values (16). Studies on the relationship between RESLES and anti-neuronal antibodies are extremely rare. It has only been described previously in cases of anti-NMDAR encephalitis, anti-VGKC encephalitis, anti-GFAP encephalitis, and anti-Yo encephalitis, respectively (34–37). A patient presented with both RESLES and auto-antibodies against voltage-gated potassium channels; the authors suggested a potential causal role for voltage-gated potassium channel autoantibodies, causing changes in potassium concentration within the myelin sheath (37). This may cause hyperpolarization, increased ion concentration, and excitotoxicity, ultimately leading to intramedullary edema manifesting as splenic lesions with diffusion restriction on MRI. A previous study found differences in fractional anisotropy (a measure of diffusion in MRI) in the SCC of patients with systemic lupus, which might be a relationship between more general autoimmune processes and changes detectable by diffusion imaging (38). In addition, the reason why the SCC is a favored site remains unknown. This may be because the enriched vascular supply of SCC, mainly provided by the vertebrobasilar system, could have resulted in increased delivery of auto-antibodies to this region and brought about an inflammatory infiltrate, causing the lesion of SCC (39). A previous case reported RESLES in a patient with anti-NMDAR antibody encephalitis, but the exact mechanism was unknown. In contrast, our case showed overlapping auto-antibodies with RESLES, and repeated CSF anti-NMDAR autoantibodies returned to normal.

The patient in this case had an excellent treatment response. Therefore, her complete recovery from cognitive impairment, the fact that her daily life remained significantly unaffected, and that she resumed normal work constitute a significant strength of this study. Nevertheless, the study has some limitations. First, this is a case study of one individual and therefore no generalizable conclusions or recommendations may be adopted. Secondly, the patients failed to undergo timely surgical treatment for personal reasons, increasing tumor size. Although the patient recovered extremely well, we did not perform a fourth serum anti-NMDAR antibodies test to establish whether the titer of antibodies decreased to normal levels.

In conclusion, this was a rare case of AE with overlapping auto-antibodies, along with RESLES and bilateral ovarian teratomas. The current case hints a possibility of the concurrence of mGluR5 antibodies in anti-NMDAR encephalitis, however, the underlying mechanism remains elusive. We suggest that anti-NMDAR antibodies may be mechanistically linked to the RESLES as described above. Nevertheless, the possibility that anti-mGluR5 autoantibodies might cause RESLES cannot be excluded. Furthermore, we provide additional evidence that overlapping antibodies-related pathology may be one of the many causes of RESLES. However, caution should be observed in interpreting the observation, considering that this is a single-case study. Therefore, additional future studies with a large number of cases may be necessary to improve the understanding of RESLES.

## Data availability statement

The original contributions presented in the study are included in the article/**Supplementary Material**. Further inquiries can be directed to the corresponding author.

## Ethics statement

Written informed consent was obtained from the individual(s) for the publication of any potentially identifiable images or data included in this article.

## Author contributions

YL wrote the case report under the guidance of MZ. TY and MZ are the Neurologist who treated the patient. ZW is the Gynecologist who treated the patient. MW, DL, and JS from radiology were responsible for the interpretation of images. SX from pathology was responsible for the interpretation of images. All authors contributed to the article and approved the submitted version.
