# Supplementary figures and images for: Case report: Autoimmune encephalitis with multiple auto-antibodies with reversible splenial lesion syndrome and bilateral ovarian teratoma
Source: Front Immunol. 2023 Jan 12;13:1029294. doi: 10.3389/fimmu.2022.1029294 (PMC9878315; doi:10.3389/fimmu.2022.1029294)

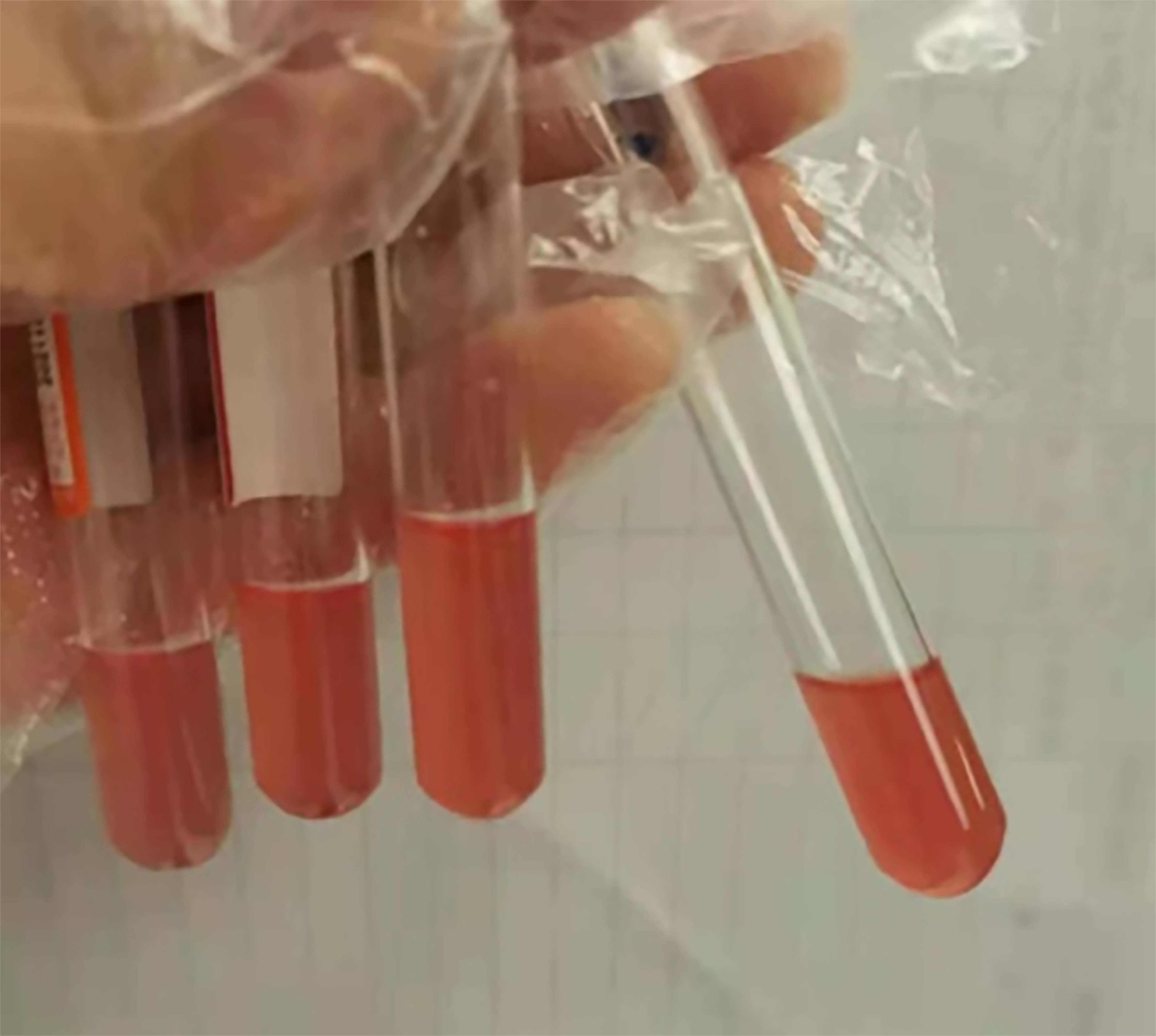

Supplement: Supplementary file 1 [file Image_1.jpeg]
